# Impact of health education intervention on knowledge and perception of cervical cancer and screening for women in Ghana

**DOI:** 10.1186/s12889-019-7867-x

**Published:** 2019-11-11

**Authors:** Nancy Innocentia Ebu, Salome Amissah-Essel, Christiana Asiedu, Selorm Akaba, Kingsley Asare Pereko

**Affiliations:** 1 Department of Adult Health, School of Nursing and Midwifery, University of Cape Coast, Cape Coast, Ghana; 2 Department of Health, Physical Education and Recreation, Faculty of Science and Technology Education, University of Cape Coast, Cape Coast, Ghana; 3 Department of Community Medicine, School of Medical Sciences, University of Cape Coast, Cape Coast, Ghana; 4 Department of Agricultural Economics and Extension, School of Agriculture, University of Cape Coast, Cape Coast, Ghana

**Keywords:** Cervical cancer, Education intervention, Women, Knowledge, Health belief model, Pre-post-test, Ghana., Trial registry: ISRCTN Registry., Trial registration: Current Controlled Trials ISRCTN13468193., Date: 22/03.2019.

## Abstract

**Background:**

The burden of cervical cancer continues to rise in developing economies. Women in the sub-Saharan African region have higher chances of developing cervical cancer due to a greater prevalence of related risk factors. The purpose of this study was to determine the effect of health education intervention on cervical cancer and screening perceptions of women in the Komenda, Edina, Eguafo, and Abirem (K.E.E.A) District in the Central Region of Ghana.

**Methods:**

A non-equivalent control-group design was used to select church women; 396 in the intervention group and 386 in the control group, aged 11 to 70 years in the K.E.E.A District in the Central Region of Ghana. Data was collected via a validated structured interview schedule and analysed using the paired - and independent-samples t-tests, Kruskal-Wallis test, and Mann-Whitney U test.

**Results:**

A comparison of the mean differences between the pre-post-test scores for the intervention and control groups showed a statistically significant difference for knowledge of cervical cancer (t = 6.22, df = 780, *p* = 0.001), knowledge of cervical cancer screening (t = 5.96, df = 780, *p* = 0.001), perceived seriousness (t = 3.36, df = 780, *p* = 0.001), perceived benefits (t = 9.19, df = 780, *p* = 0.001), and perceived barriers (t = 3.19, df = 780, *p* = 0.001). However, perceived susceptibility for the intervention group reduced, evidenced by a decrease in the mean (mean = − 0.12) compared to the control group (mean = 0.93) and this was statistically significant (t = 2.72, df = 780, *p* = 0.007).

**Conclusions:**

Health education interventions are critical in improving knowledge and perceptions, and increasing self-efficacy of women about cervical cancer and screening.

## Background

Cervical cancer is a disease of concern to women’s health worldwide. It is estimated that 8.6 million women above 15 years of age in Ghana are at increased risk of developing cervical cancer [1]. In addition, annually, 3052 women are diagnosed with cancer of the cervix with some 1556 deaths occurring in Ghana [1]. Cancer of the cervix is caused by the Human Papilloma Virus (HPV) which is a sexually transmitted infection (STI) [2]. Infection with the HPV could take about 15 to 20 years before leading to cervical cancer, especially in individuals with normal immune function [2]. The disease can be prevented through vaccination and screening and treatment of precancerous lesions, and Ghana commenced implementation of HPV vaccination in 2013 [3].

Although cervical cancer can be prevented through early screening and treatment of precancerous lesions, cervical cancer screening in Ghana seems to have been restricted to the regional and teaching hospitals as well as some few private health facilities, and most women at the community level lack access to cervical cancer screening services [3]. The low level of awareness and knowledge about the disease and screening are some of the factors impacting cervical cancer screening utilisation [4, 5]. A study conducted in Elmina showed that only 6.4% of women had knowledge about cervical cancer and 2.3% had knowledge about Pap smear tests [6].

Therefore, efforts to increase awareness, knowledge and understanding of the perceptions of women about cervical cancer and screening through the provision of an educational intervention will be an important step in promoting the health of women. Health education may enable women to increase their intention to screen. Evidence from a systematic review of studies conducted in developed settings strongly supports the use of health education programmes in increasing cervical cancer screening utilisation [7]. Nonetheless, in Ghana, there seems to be a paucity of data on the impact of health education intervention on cervical cancer screening utilisation. This study is meant to fill a gap in the existing literature in the area of promoting women’s health. The study hypothesised that there would be an increase in knowledge about cervical cancer, knowledge about cervical cancer screening, perceived susceptibility, perceived seriousness, perceived benefits, and self-efficacy in the intervention group compared to the control group. This study also hypothesised that perceived barriers about cervical cancer screening would decrease for women in the intervention group compared to the control group.

## Methods

### Research Design

The study was conducted in the Komenda, Edina, Eguafo, Abirem (K.E.E.A.) District in the Central Region of Ghana using a non-equivalent control group design. By using this design, there was a possibility of selection bias which was mitigated by carefully and randomly selecting participants in both the intervention and control groups.

### Population and Setting

The population of women was estimated as 22,064 [8]. Women in this study referred to female adolescents and adults. The K.E.E.A District which is located in the southern part of Ghana was selected because most girls indulge in sexual activities before attaining 15 years of age without using a condom, which may put them at risk of HPV and cervical cancer [8, 9]. This calls for an urgent need to provide educational intervention about cervical cancer and screening. The indigenes of K.E.E.A are mostly involved in fishing and farming. There are health centres in the district that offer primary health care [10]. However, these health facilities do not provide cervical cancer screening services. The nearest health facilities that offer cervical cancer screening are the Cape Coast Teaching Hospital and a private medical laboratory, Life Sciences Diagnostic Centre, all in the Cape Coast Metropolis which is about 12 km from the capital of the K.E.E.A District, Elmina.

### Sample and Sampling

Considering the population of women in the K.E.E.A District (22,064), the study estimated 394 participants using the equation proposed by Glenn [11]. The parameters considered were precision level of plus or minus 5%, confidence level of 95% and a proportion of 50% which reflects the maximum variability in the population [11]. A sample size of 394 was required for this study, as this would be representative of the total population of women in the district. This was increased by 6% to cover any degree of uncertainties such as dropouts. Therefore, the total sample size required was 418 each for intervention and control groups. However, due to attrition and migration, a total sample size of 396 was realized for the intervention group and 386 for the control group.

Although the Ministry of Health’s guidelines on non-communicable diseases stipulate cervical cancer screening for women 25-64 years [12], women aged 11 to 70 years residing in the K.E.E.A District were included in the eligibility criteria for this study. It was assumed that if women as young as 11 years obtain information about cervical cancer and screening, they would be well informed about the risk factors and prevention strategies in reducing their vulnerability.

Out of a total of five major towns in the district - Elmina, Komenda, Eguafo, Abirem and Kissi - two were randomly selected using simple random sampling with replacement technique and these constituted the intervention and control groups. Elmina Township was selected as the intervention group whilst Kissi constituted the control group.

The criteria for selection of churches for inclusion in the study were that the church should have regular congregation and have a branch in Elmina and Kissi. Using this criteria, twelve churches were included in the study. Out of the twelve churches found in both towns, eight were randomly selected to participate in the study using the simple random sampling with replacement technique. It was assumed that selection of the eight churches will provide adequate sample for the study.

The justification for using churches is that Ghanaian women believe in faith for healing and most people rely on supernatural forces for healing, which could affect the rate of detection of diseases and its outcome [13, 14]. This strongly suggests that women in churches will be more likely to cooperate and this could also encourage participation in cervical cancer screening [15, 16]. The findings of this study may however not be generalised to all women because the characteristics of women involved in this study may differ from those who belong to other religious sects or social gatherings.

The congregation sizes for the churches differed and there was no valid list for all members of the varied churches. Therefore, participants from the selected churches were conveniently sampled based on their consent to participate in the study. In all churches, eligible women interested in participating were enrolled in the study. However, there was a dropout rate of 5.3% in the intervention group and 7.7% in the control group.

### Data Collection

A questionnaire was adapted from Ebu et al. [6] and Mupepi et al. [5]. The adaptation was to make it culturally relevant and applicable to the current study based on the hypotheses the study sought to test. The outcome measures of the study were knowledge of cervical cancer, knowledge of cervical cancer screening, perceived susceptibility, perceived seriousness, perceived benefits, perceived barriers, and self-efficacy. Perceived susceptibility was operationally defined as an individual’s subjective perception of being at risk of cervical cancer, perceived seriousness – subjective evaluation of an individual to perceive the seriousness or the possible consequences of cervical cancer, and perceived barriers - factors perceived to be hindering one’s ability to engage in cervical cancer screening or overcoming possible factors associated with seeking cervical cancer screening. Perceived benefit was defined as the subjective benefits of engaging in cervical cancer screening to prevent cervical cancer which is influenced by an individual’s level of motivation, and self-efficacy – the confidence to engage in cervical cancer screening. They were all measured at baseline and 6 weeks. The method of measurement for all the outcome measures was a questionnaire. The instrument had already been validated with the following Cronbach’s alphas: knowledge about cervical cancer = .738, knowledge about cervical cancer screening = .704, perceived susceptibility = .824, perceived seriousness = .820, perceived benefits = .798 and perceived barriers = .795. Face and content validity were achieved by showing it to experts in the field of cervical cancer and screening.

The following constructs of the Health Belief Model were used to determine women’s knowledge and perception of cervical cancer and screening. Knowledge about cervical cancer consisted of 10 items on the risk factors, signs and symptoms, and prevention. Knowledge about cervical cancer screening consisted of five items. Perceived susceptibility, perceived seriousness, and perceived barriers subscales consisted of eight items each. Perceived benefits subscale consisted of five items. The items on knowledge about cervical cancer and screening were categorical variables with the responses “Yes”, “No” and “Don’t Know”. Perceived susceptibility, perceived seriousness, perceived benefits, and perceived barriers were measured on a four-point Likert scale.

Participants were recruited from churches by making announcements about the project in the respective churches. Six diploma prepared nurses were trained on the use of the standardised educational tool to deliver effective health education on cervical cancer and screening. They also received training on how to administer the questionnaire to both the intervention and the control groups in order to obtain relevant data for the study. This was delivered through face to face interview with the individual respondents. For both the intervention and control groups, an initial pre-test data were collected from eligible participants who volunteered for the study. This was followed by education on cervical cancer and screening in the intervention group.

### Intervention

The intervention comprised a comprehensive education on cervical cancer and screening. Women in the selected churches in Elmina constituted the intervention group. Women from the selected churches who consented to participate in the education intervention were grouped into their various churches and assessed before giving health education using a standardised educational tool on cervical cancer and screening. The health education included information on cervical cancer and screening to increase the level of awareness about the disease. The education on cervical cancer focused on the cause, predisposing factors, signs and symptoms, complications and methods of prevention. Regarding cervical cancer screening, participants were introduced to where they could go for testing, persons who carry out the test and the part of the body needed for the test. The benefits of cervical cancer screening and the perceived barriers to screening were addressed. This educational intervention spanned a period of six weeks and included the use of lectures, discussions, videos, and leaflets. The health education was given once every week in the respective churches. Therefore, each participating church had six sessions. Each of the sessions were delivered by a qualified nurse with sufficient knowledge about the disease. On average, each lecture took approximately 1 hour after which participants were given the opportunity to ask questions and any misconceptions clarified. Participants were also given time to reflect and discuss issues concerning cervical cancer and screening amongst themselves. After six weeks of education, the participants were reassessed with the same instrument as used prior to the educational intervention to assess any changes in knowledge and perceptions about the disease and screening. For participants who agreed to participate in the study but due to some reasons were not available at the time of data collection or the intervention, efforts were made to either collect the data or carry out the health education at a later date convenient to them but within the time for the project.

For the control group, initial data were collected from eligible women in the churches constituting the control group who volunteered to participate in the study. Participants from the selected churches were conveniently sampled based on their consent to participate in the study. Data were collected from the same group six weeks after the initial data collection.

### Data Analysis

Measurement of knowledge about cervical cancer consisted of 10 items on a dichotomous scale. Each correct answer on the items was assigned a score of one and an incorrect answer attracted a score of zero. The individual scores were computed for pre- and post-tests in both groups and used for the analysis. Knowledge about cervical cancer screening consisted of 5 items. The items were also dichotomous and scored and computed as described for knowledge on cervical cancer. The perception aspect of the questionnaire contained questions on perceptions of susceptibility, seriousness, benefits and barriers. These were scored by participants strongly agreeing, agreeing, disagreeing or strongly disagreeing to each of the statements that constituted the sub-scales. For positive statements on the sub-scale, strongly agree was assigned a score of (4), agree (3), disagree (2) and strongly disagree (1). The reverse score was used for negative statements. The individual scores for perceived susceptibility, seriousness, benefits and barriers were computed for the levels of agreement before and after the intervention in both groups and used for the analysis. Self-efficacy was measured using a single item with a binary outcome of how confident they are in seeking cervical cancer screening.

Data were analysed with the Statistical Package for Social Sciences software version 21.0 (IBM Corporation, Armonk, NY, USA). The paired sample t-test was used to determine knowledge about cervical cancer, knowledge about cervical cancer screening, perceived susceptibility, perceived seriousness, and perceived benefits within the intervention group by comparing the before and after intervention scores. A similar analysis was done for the control group. The independent-sample t-test was used to determine the effect of the intervention by comparing participants scores on knowledge about cervical cancer, knowledge about cervical cancer screening, perceived susceptibility, perceived seriousness, and perceived benefits between the intervention and control groups. Self-efficacy for the various participants before and after the educational intervention were also analysed using the Kruskal-Wallis test with the Post Hoc performed using Mann-Whitney U.

## Results

### Socio-demographic Characteristics of the Respondents

Table 1 presents the socio-demographic characteristics of the respondents in both the intervention and control groups. For the intervention group, 21.5% of the women were within the age group 50-59 years. Regarding marital status,44.4% of the women in the intervention group were married, compared 52.6% in the control group. In connection with the educational status of the women, 45.2% of the women in the intervention group had primary education whiles 50.0% of the women in the control group had primary education. Also, 17.7% of women in the intervention group had no formal education. Table 1 further shows that a higher percentage of women in the intervention group, 70.7%, were employed. It is worth mentioning that women in this community are mostly self-employed. They are basically fish mongers, farmers and petty traders.

**Table 1:** Socio-demographic Distribution of Respondents

Selected variables	Communities		Total
	Elmina	Kissi	
Age			
10–19	10.6	14.5	12.5
20–29	16.7	21.0	18.8
30–30	18.7	20.7	19.7
40–49	21.0	17.9	19.4
50–59	21.5	15.8	18.7
60–69	11.6	9.6	10.6
Marital status			
Single	34.6	29.3	32.0
Married	44.4	52.6	48.5
Divorced	9.3	8.0	8.7
Widowed	9.6	8.5	9.1
Cohabiting	2.0	1.6	1.8
Level of Education			
No formal education	17.7	22.3	19.9
Primary	45.2	50.0	47.6
Secondary	26.0	20.5	23.3
Tertiary	11.1	7.3	9.2
Employment status			
Retired	2.8	1.0	1.9
Student	13.6	11.9	12.8
Unemployed	12.9	16.1	14.5
Employed	70.7	71.0	70.8
Total	100.0	100.0	100.0

N for Kissi = 386, N for Elmina = 396

Intervention Control

### Paired samples t-test on Pre- and Post-test Education Intervention on Cervical Cancer and Screening for Women in the intervention group

Table 2 presents the results of a paired-samples t-test conducted to compare the effect of cervical cancer and screening education on women’s knowledge of cervical cancer, knowledge of cervical cancer screening, perceived susceptibility, perceived seriousness, perceived benefits, and perceived barriers in the intervention group. As can be seen in Table 2, a comparison of the mean for knowledge of cervical cancer before (mean = 3.44) and after (7.12) the intervention shows that there might be some difference. To test whether the difference in mean between the two conditions was statistically significant, a paired samples t-test was conducted. The result of this test revealed that there was a statistically significant difference between the pre and posttest (t = 25.25, df = 395, *p* = 0.001).

**Table 2:** Paired samples t-test on Pre- and Post-test Education Intervention on Cervical Cancer and Screening for Women in the intervention group

Variables		N	Mean	Std. Dev.	Mean Difference	T	df	*P*-value
Knowledge of cervical cancer	Before	396	3.44	2.19	3.67	25.25	395	0.001
	After	396	7.12	1.91				
Knowledge of cervical cancer screening	Before	396	2.49	1.07	1.11	15.62	395	0.001
	After	396	3.59	0.94				
Perceived susceptibility	Before	396	18.37	3.75	0.12	0.44	395	0.331
	After	396	18.24	4.06				
Perceived seriousness	Before	396	23.94	3.88	2.26	8.93	395	0.001
	After	396	26.20	3.46				
Perceived benefits	Before	396	20.39	2.66	1.50	8.13	395	0.001
	After	396	21.89	2.77				
Perceived barriers	Before	396	20.57	3.92	0.96	3.46	395	0.001
	After	396	21.54	3.78				

Also, comparison of the mean for knowledge of cervical cancer screening, perceived seriousness, perceived benefits and perceived barriers before and after the intervention in Table 2 shows some differences. These were also tested statistically using paired samples t-tests and there was a statistically significant difference between the pre and posttest scores for knowledge of cervical cancer screening (t = -15.62, df = 395, *p* = 0.001), perceived seriousness (t = 8.93, df = 395, *p* = 0.001), perceived benefits (t = 8.13, df = 395, *p* = 0.001), and perceived barriers (t = 3.46, df = 395, *p* = 0.001). However, there was no statistically significant difference between the pre and posttest scores for perceived susceptibility (t = 0.44, df = 395, *p* = 0.331).

### Paired samples t-test on Pre and Posttest Cervical Cancer and Screening for Women in the Control Group

Table 3 presents the results of the paired-samples t-test performed on women’s cervical cancer and screening pre and posttest without any intervention. Comparison of the mean for the two conditions for knowledge of cervical cancer, knowledge of cervical cancer screening, perceived susceptibility, perceived seriousness, and perceived benefits shows some differences. The differences were tested statistically, and there was a statistically significant difference between the before and after the test for knowledge of cervical cancer (t = 16.09, df = 385, *p* = 0.001), knowledge of cervical cancer screening (t = 4.75, df = 385, *p* = 0.001), perceived susceptibility (t = 3.53, df = 385, *p* = 0.001), perceived seriousness (t = 3.37, df = 385, *p* = 0.001), and perceived benefits (t = 4.96, df = 385, *p* = 0.001). However, there was no statistically significant difference for the two conditions for perceived barrier (t = 0.99, df = 385, *p* = 0.162).

**Table 3:** Paired samples t-test on Pre and Posttest Cervical Cancer and Screening for Women in the Control Group

Variables		Mean	N	Std. Dev	Mean Difference	T	df	P-value
Knowledge cervical cancer	Before	2.53	386	2.24	2.38	16.09	385	0.001
	After	4.91	386	2.01				
Knowledge cervical cancer screening	Before	2.43	386	1.04	0.43	4.75	385	0.001
	After	2.86	386	1.49				
Perceived susceptibility	Before	18.00	386	3.71	0.93	3.53	385	0.001
	After	18.93	386	4.24				
Perceived seriousness	Before	22.99	386	4.32	0.97	3.37	385	0.001
	After	23.96	386	3.62				
Perceived benefits	Before	20.37	386	2.72	0.97	4.96	385	0.001
	After	19.39	386	2.98				
Perceived barrier	Before	20.17	386	3.31	0.26	0.99	385	0.162
	After	19.91	386	3.82				

### Independent samples t-test on Pretest Scores on Cervical Cancer and Screening for the Intervention group (Elmina) and control group (Kissi)

Table 4 presents the results of the independent-samples t-test performed on pretest scores for cervical cancer and screening of two independent groups of women in the intervention and control groups. The baseline information of the women about cervical cancer and screening in both groups were assessed. As can be seen in Table 4, comparison of the mean for knowledge of cervical cancer in the two independent groups suggests that women in the intervention group had more information about cervical cancer (mean = 3.44) compared to the control group (mean = 2.53). There was also a difference in the mean for perceived seriousness in the two groups. Women in the intervention group had a higher mean (mean = 23.94) compared to the control group (mean = 22.99). To test whether the difference in mean between the two groups was statistically significant, the independent-samples t-test was performed. The results of the t-test revealed that there was a statistically significant difference in knowledge of cervical cancer (t = 5.78, df = 777.931, *p* = 0.001) and perceived seriousness (t = 3.24, df = 780, *p* = 0.001) in the two groups. However, there was no statistically significant difference in the two independent groups for knowledge of cervical cancer screening (t = 0.76, df = 780, *p* = 0.224), perceived susceptibility (t = 1.38, df = 780, *p* = 0.084), perceived benefits (t = 0.11, df = 780, *p* = 0.457) and perceived barriers (t = 1.54, df = 764.656, *p* = 0.062).

**Table 4:** Independent samples t-test on Pretest Scores on Cervical Cancer and Screening for the Intervention group (Elmina) and control group (Kissi)

Variables	Categories	N	Mean	Std. Dev.	Mean Difference	t	df	*P*-value
Knowledge of cervical cancer	Elmina	396	3.44	2.19	0.92	5.78	777.931	0.001
	Kissi	386	2.53	2.24				
Knowledge of cervical cancer screening	Elmina	396	2.49	1.07	0.06	0.76	780	0.224
	Kissi	386	2.43	1.04				
Perceived susceptibility	Elmina	396	18.37	3.75	0.37	1.38	780	0.084
	Kissi	386	18.00	3.71				
Perceived seriousness	Elmina	396	23.94	3.88	0.95	3.24	780	0.001
	Kissi	386	22.99	4.32				
Perceived benefits	Elmina	396	20.39	2.66	0.02	0.11	780	0.457
	Kissi	386	20.37	2.72				
Perceived barriers	Elmina	396	20.57	3.92	0.39	1.54	764.656	0.062
	Kissi	386	20.17	3.31				

### Independent samples t-test on Posttest Scores on Cervical Cancer and Screening for the Intervention group (Elmina) and Control group (Kissi)

Table 5 presents the results of the independent-samples t-test performed on posttest scores for cervical cancer and screening of two independent groups of women in the intervention and control groups. Table 5 shows differences in the intervention and control groups on knowledge of cervical cancer (mean = 2.21), knowledge of cervical cancer screening (mean = 0.74), perceived susceptibility (mean = 0.68), perceived seriousness (mean = 2.24), perceived benefits (mean = 2.49) and perceived barriers (mean = 1.63). To test whether the mean differences between the posttest scores for the intervention and control groups were statistically significant, the independent-samples t-test was conducted. The results revealed that there was a statistically significant difference in the mean for knowledge of cervical cancer (t = 15.76, df = 780, *p* = 0.001), knowledge of cervical cancer screening (t = 8.29, df = 646.765, *p* = 0.001), perceived susceptibility (t = 2.30, df = 780, *p* = 0.022), perceived seriousness (t = 8.85, df = 780, *p* = 0.001), perceived benefits (t = 12.11, df = 780, *p* = 0.001), and perceived barriers (t = 5.98, df = 780, *p* = 0.001).

**Table 5:** Independent samples t-test on Posttest Scores on Cervical Cancer and Screening for the Intervention group (Elmina) and Control group (Kissi)

Variables	Categories	N	Mean	Std. Dev	Mean Difference	t	df	*P*-value
Knowledge of cervical cancer	Elmina	396	7.12	1.91	2.21	15.76	780	0.001
	Kissi	386	4.91	2.01				
Knowledge of cervical cancer screening	Elmina	396	3.59	0.94	0.74	8.29	646.765	0.001
	Kissi	386	2.86	1.49				
Perceived susceptibility	Elmina	396	18.24	4.06	0.68	2.30	780	0.022
	Kissi	386	18.93	4.24				
Perceived seriousness	Elmina	396	26.20	3.46	2.24	8.85	780	0.001
	Kissi	386	23.96	3.62				
Perceived benefits	Elmina	396	21.89	2.77	2.49	12.11	780	0.001
	Kissi	386	19.39	2.98				
Perceived barriers	Elmina	396	21.54	3.78	1.63	5.98	780	0.001
	Kissi	386	19.91	3.82				

### Independent-sample t-test between the differences of the pre-post-test of the intervention and control groups

Table 6 presents the Independent-sample t-test between the differences of the pre-test-post-test results of the intervention and control groups. The intervention group received education on cervical cancer and screening while the control group did not have any form of education on cervical cancer. A comparison of the mean differences between the pre-post-test of the intervention and control groups suggests a higher mean for knowledge of cervical cancer in the intervention group (mean = 3.67) compared to the control group (mean = 2.38). The mean difference was statistically significant for knowledge of cervical cancer (t = 6.22, df = 780, *p* = 0.001). Again, the intervention group had higher mean for knowledge of cervical cancer screening (mean = 1.11), perceived seriousness (mean = 2.26), perceived benefits (mean = 1.50) and perceived barriers (mean = 0.96) compared to the control group (0.43, 0.97, -0.97 and -0.26, respectively). These differences were tested using the independent-samples t-test, and findings revealed statistically significant mean difference for knowledge of cervical cancer screening (t = 5.96, df = 780, *p* = 0.001), perceived seriousness (t = 3.36, df = 780, *p* = 0.001), perceived benefits (t = 9.19, df = 780, *p* = 0.001), and perceived barriers (t = 3.19, df = 780, *p* = 0.001). However, perceived susceptibility for the intervention group reduced, evidenced by a decrease in the mean (mean = -0.12) compared to the control group (mean = 0.93) and this was statistically significant (t = 2.72, df = 780, *p* = 0.007).

**Table 6:** Independent-sample t-test between the differences of the pre-post-test of the intervention and control groups

Variables	Categories	N	Mean	Std. Dev.	Mean Difference	t	df	*P*-value
Difference in knowledge on cervical cancer	Elmina	396	3.67	2.89	1.29	6.22	780	0.001
	Kissi	386	2.38	2.91				
Difference in knowledge on screening	Elmina	396	1.11	1.42	0.68	5.96	736.38	0.001
	Kissi	386	0.43	1.77				
Difference in perceived susceptibility	Elmina	396	−0.12	5.62	1.05	2.72	780	0.004
	Kissi	386	0.93	5.157				
Difference in perceived seriousness	Elmina	396	2.26	5.04	1.29	3.36	780	0.001
	Kissi	386	0.97	5.68				
Difference in perceived benefits	Elmina	396	1.50	3.68	2.47	9.19	780	0.001
	Kissi	386	−0.97	3.85				
Difference in perceived barriers	Elmina	396	0.96	5.54	1.23	3.19	780	0.001
	Kissi	386	−0.26	5.20				

Furthermore, the pre-post-test scores for the two groups were assessed on the basis of their self-efficacy. Kruskal-Wallis test performed on the data obtained showed that there was a statistically significant effect of health education on self-efficacy (*H* = 81.99, df = 3, *p* = 0.001). Post Hoc comparisons using the Mann-Whitney U test with Bonferronni correction at 0.0167 level of significance showed that women in the intervention group had higher self-efficacy (mean rank = 435.77) compared to the control group (mean rank = 346.08).

## Discussion

### Effects of Health Education Intervention on Knowledge of Cervical Cancer and Cervical Cancer Screening

The findings of the study suggest an increase in knowledge about cervical cancer in the intervention group compared to the control group, evidenced by a higher mean for the intervention. A possible explanation of this finding may be that the participants in the intervention group may have gained some knowledge after they had been educated about the disease. This supports the findings of a health education intervention study on cervical cancer conducted in Nigeria, Jamaica and Egypt [17–21]. In Nigeria, the intervention increased the level of knowledge and awareness of cervical cancer and screening [22]. A similar finding was observed in Jamaica as participants had a massive improvement in knowledge about cervical cancer risk factors, symptoms and prevention [20]. Cervical cancer intervention programme for married women in Egypt also reported a significant improvement in knowledge about the disease after the intervention. These studies highlight the important role health education plays in shaping knowledge of health appropriate behaviours. Interestingly, although the control group did not receive any education, there was a marginal increase in knowledge of cervical cancer between the pretest and the posttest scores within this group. This suggests that the pretest might have motivated participants to search for information on cervical cancer.

The health education intervention was observed to have impacted knowledge of cervical cancer screening, as the intervention group had higher scores after the intervention compared to the control group. A possible explanation for this observation may be that the participants had comprehensive information about screening during the education sessions. The finding of the present study is consistent with the findings of previous intervention studies [17, 20, 21]. The similarities in the methodology employed could account for the findings observed. A comparison of the post-test scores for the intervention and control groups strongly suggests that the health education intervention enhanced the knowledge of women about cervical cancer screening. Surprisingly, a critical examination of the pre and post-test scores for knowledge of cervical cancer screening in the control group points to the fact that even without any intervention, there was a slight improvement in knowledge. Since the study was a community-based one, certain factors were difficult to control, so participants might have read or obtained information about cervical cancer screening after the initial assessment.

### Effects of Health Education Intervention on Perceptions of Cervical Cancer Screening

The findings further suggest that perceived seriousness about cervical cancer increased for women in the intervention group compared to the control group after the pre-post-test scores for both the intervention and control groups have been compared. This indicates that the health education might have enabled participants to evaluate the complications associated with the disease and how these could impact their health and well-being, as evidenced by a higher mean for the post-test scores for the intervention and the differences between the pre-post-test scores for the intervention compared to the control group. An earlier study found health education to have improved perception of the seriousness of cervical cancer [21]. The homogeneity in the methodologies between the current study and that of Ahmed et al. [21] could explain the similarity in findings. Nonetheless, in studies conducted among Turkish and incarcerated women in the United States, perceived susceptibility was low for women in the intervention group [22, 23]. Cultural sensitivity and level of literacy among these populations may have accounted for the differences in findings. Again, differences in the health education tool used for the intervention in these studies may have contributed to the observed findings. Furthermore, a critical analysis of the findings suggests that within the intervention group only, the level of perceived susceptibility actually decreased whilst it increased in the control group. A possible explanation could be that health education enabled women in the intervention group to evaluate their level of risk about cervical cancer. It could also be that since they are now equipped with adequate information about the risk factors, they are more prepared to adopt measures that will protect them from getting the disease.

In addition, perceived benefits of cervical cancer screening significantly increased for women in the intervention group compared to the control group after the pre-post-test assessment. This finding indicates that the participants may have understood the benefits of engaging in cervical cancer screening as a result of the health education intervention. Earlier studies affirmed this finding [21, 24, 25]. A previous study reported that health education changed women’s perception of the benefits of preventing cancer of the cervix and modified their beliefs [24]. Similarly, an educational programme conducted for married women in Egypt saw a greater improvement in perceived benefits of cervical cancer screening after the programme [21]. In Nigeria, peer education greatly influenced the perception of the benefits of cervical screening [25]. It is worth mentioning that within the intervention group only, there was an increase in the perception of the benefits of screening after comparing the before and after scores. However, the after scores for the control group significantly decreased. It could be assumed that the education intervention contributed to the difference since the control group did not receive any form of education; the participants in the control group may have provided responses that put them in good light.

The study’s findings failed to support the hypothesis that perceived barriers about cervical cancer screening will decrease for women in the intervention group compared to the control group as evidenced by a higher mean for the intervention group after comparing the differences between the pre-post-test scores. Additionally, comparison of the post-test scores for the intervention and control group suggests that the intervention group had more barriers compared to the control group. A possible explanation could be that health education exposed the women in the intervention group to the reality of the problem of cervical cancer by enlightening them on the challenges to seeking cervical cancer screening. Additionally, the intervention took place in a fishing community, and most of the women have low socio-economic status, as they are mainly into petty trading [10]. The finding of the current study is consistent with that described by Ahmed et al. [21] in which the perception of barriers was found to be high after the implementation of the intervention among married women in Egypt. In contrast, other studies conducted in developed settings have reported fewer barriers post-intervention within the intervention group [22, 24]. It is plausible to assume that in developed settings, there may be well-structured programmes to facilitate cervical cancer screening, so women may not encounter many challenges in an attempt to have a screening test done. However, in resource-constrained settings like Ghana, cervical cancer screening facilities may not be well developed [4].

Furthermore, the findings of the study failed to support the hypothesis that perceived susceptibility to cervical cancer will increase for women in the intervention group compared to the control group. It seems the women were able to evaluate their level of risk as a result of the health education programme and found out to be less at risk. A possible explanation could also be that the participants gave socially desirable responses since they are from religious groups and that may have affected the outcome of the study. Similar findings were reported by Bebis et al. [23] among Turkish women and Ramaswamy et al. [22] among incarcerated women in the United States in which the after-intervention scores demonstrated less susceptibility to cervical cancer. In the present study, perceived susceptibility scores were found to be high for the control group when the posttest scores for the intervention and control groups were compared. Again, the findings suggest that the control group had higher susceptibility perception after comparing pre-post-test scores for both the intervention and control groups. It could be assumed that women in the control group provided responses that put them in good light. It could also be possible that participants in the control group may have searched for information about the disease, which could have influenced the outcome of their responses. Nonetheless, other empirical works strongly suggest that perception of susceptibility significantly increased after intervention in Egypt, Greece, and Nigeria [21, 24, 25]. Therefore, the effect of health education on perceived susceptibility is unclear in the literature, which requires further evidence.

Additionally, self-efficacy or level of confidence is an important determinant of health behaviour [26, 27]. Several studies have examined its impact on cervical cancer screening [27–29]. The findings of the present study suggest that women who were educated on cervical cancer and screening had higher self-efficacy. This finding is consistent with the findings of an intervention study conducted in Turkey in which levels of self-efficacy greatly improved in the intervention group compared to the control group [28]. Women with increased self-efficacy may have a higher tendency of engaging in health appropriate behaviours, since they may have been exposed to some information that may have influenced their knowledge status. It is critical to note that direct experience of mastery can greatly increase self-efficacy belief [30]. An earlier study reported a direct relationship between knowledge level [31], health literacy [32], and self-efficacy. Education intervention study among diabetes patients reported increased levels of self-efficacy after the education sessions [33]. Additionally, a randomised controlled trial reported that needs-based client education may significantly improve the level of confidence and health status [34]. This implies that self-efficacy is critical in enabling individuals to successfully perform actions that can potentially improve their health.

## Conclusions

The findings of the study suggest that health education, which employed lectures, discussions, videos, and leaflets, may be critical in shaping knowledge of cervical cancer and screening, changing perceptions, and building self-efficacy towards cervical cancer screening in Ghana. In the present study, the intervention group demonstrated high knowledge and positive beliefs towards cervical cancer screening despite the barriers to cervical cancer screening. It was evident that the barriers could hinder eligible women from seeking screening, though they may be well informed and have increased self-efficacy. Measures to reduce the barriers may increase cervical cancer screening uptake among the study population. It is critical to note that education interventions may enable women to evaluate their level of susceptibility and take measures to reduce risks of contracting the disease, as those who received the health education in this study self-reported to be less susceptible to cervical cancer. The findings of the study are important in guiding health education interventions on cervical cancer and screening in Ghana.

## Data Availability

The datasets from which the results were generated are not in the public domain. It will be made available on reasonable request from the corresponding author. However, permission will also be obtained from the funding agency.

## Abbreviations

HPV: Human Papilloma Virus
K.E.E.A: Komenda, Edina, Eguafo, Abirem
STI: Sexually Transmitted Infections
